# The target specificity of the RNA binding protein Pumilio is determined by distinct co-factors

**DOI:** 10.1042/BSR20190099

**Published:** 2019-06-04

**Authors:** Sumira Malik, Wijeong Jang, Song Yeon Park, Ji Young Kim, Ki-Sun Kwon, Changsoo Kim

**Affiliations:** 1 The Hormone Research Center, School of Biological Sciences and Technology, Chonnam National University, Yongbong-Dong, Gwangju-Si 500-757, South Korea; 2 Aging Research Center, Korea Research Institute of Bioscience and BioTechnology, 125 Gwahak-ro, Yuseong-gu, Daejon, South Korea; 3 School of Biological Sciences and Technology, Chonnam National University, Yongbong-Dong, Gwangju-Si 500-757, South Korea

**Keywords:** brat, mRNA stability, nanos, posttranscriptional, pumilio

## Abstract

Puf family proteins are translational regulators essential to a wide range of biological processes, including cell fate specification, stem cell self-renewal, and neural function. Yet, despite being associated with hundreds of RNAs, the underlying mechanisms of Puf target specification remain to be fully elucidated. In *Drosophila*, Pumilio – a sole Puf family protein – is known to collaborate with cofactors Nanos (Nos) and Brain Tumor (Brat); however, their roles in target specification are not clearly defined. Here, we identify Bag-of-marbles (Bam) as a new Pum cofactor in repression of *Mothers against dpp* (*mad*) mRNAs, for which Nos is known to be dispensable. Notably, our data show that Nos (but not Bam) was required for Pum association with *hunchback* (*hb*) mRNAs, a well-known target of Pum and Nos. In contrast, Bam (but not Nos) was required for Pum association with *mad* mRNAs. These findings show for the first time that Pum target specificity is determined not independently but in collaboration with cofactors.

## Introduction

The post-transcriptional regulation of gene expression can provide spatially localized protein synthesis. This process is necessary in whole embryos to give rise to the intricate expression patterns that support proper development, and, within the different parts of a single neuron, to ensure the subcellular protein localizations required for proper neural function. Post-transcriptional gene regulation is generally carried out by repressor proteins that recognize specific sequences in the 3′-UTRs of target mRNAs [1]. Genetic studies of pattern formation in *Drosophila* embryos identified *pumilio* (*pum*) and *nanos* (*nos*), both of which limit the expression of *hunchback* (*hb*) in the anterior segments of the embryo [2]. These studies also identified specific sequences—Nanos Response Elements (NREs)—in the 3′-UTR of *hb* mRNAs that are required for translation inhibition [3,4]. The repressive complex on the NREs, comprising Pum, Nos, and Brain Tumor (Brat), recruits d4EHP, Cup, or components of deadenylase, which induce either the decay of *hb* mRNAs or the inhibition of their translation [4–17].

Pum is a member of the Pum/fem-3 mRNA-binding factor (FBF) or Puf family [18–21]. The Pum Homology Domain (PUM-HD) or Puf domain of Puf proteins is composed of eight adjacent repeats, each possessing a sequence-specific RNA-binding activity [21–24]. Each Puf repeat interacts directly with one nucleotide of the eight nucleotide Pumilio Response Element (PRE) consensus sequence (i.e., 5′-UGUANAUA-3′, N = A/C/G/U) [25–27]. This PRE is present within the NRE of *hb* mRNAs and in the 3′-UTR of other Pum-associated mRNAs [5,26,28]. A crystal structure of Nos interacting with Pum revealed that Nos contacts three nucleotides, termed the Nos-Binding Site (NBS), immediately 5′ of the PRE in the *hb* NRE and Pum recognizing PRE [29]. Binding of Nos stabilizes the interaction of Pum with *hb* mRNAs [29]. Brat is a member of the TRIM (tripartite motif; RING, B-box and Coiled- coil) -NHL (NCL-1, HT2A, and LIN-41) family [30]. The Brat-NHL domain interacts with Pum and directly binds RNA at Brat Binding Sites (BBS); this interaction enhances Pum association with NREs [31–34]. Brat exhibits translational repression activity independent of Pum and Nos [33].

In addition to *hb* mRNAs, Pum, Brat, and Nos cooperate to regulate diverse mRNAs in diverse cell types. For example, Pum, Brat, and Nos regulate *paralytic* mRNAs to control the excitability of motor neurons and dendrite morphogenesis in peripheral neurons [35,36]. In other cases, however, neither Brat nor Nos are required for Pum-mediated translational repression. Pum and Nos (but not Brat) are required to inhibit *cycB* mRNAs and *mei-P26* mRNAs in pole cells and germline stem cells (GSCs) of the *Drosophila* ovary, respectively [10,14,37]. Pum and Brat (but not Nos) are required to inhibit *Mothers against dpp* (*mad*), *shnurri*, and *medea* mRNAs in the cystoblasts of the *Drosophila* ovary [38,39]. The mechanisms underlying these variations in the combinatorial requirements for the differential targeting and repression of different mRNAs remain poorly understood. In particular, the mechanisms underlying *mad* mRNA repression by Pum and Brat are incompletely understood. Here, we use a cell-based assay with the *mad* 3′-UTR to explore the mechanisms underlying *mad* mRNA repression. We hypothesized that the translational repressors Bag-of-marbles (Bam) and Bgcn may collaborate with Pum, as Bam physically interacts with Pum and Brat [40]. Here, we show that Bam and Bgcn do in fact collaborate with Pum and Brat in translational repression via the *mad* 3′UTR. Notably, we find that Pum binding to *mad* mRNAs in cells requires Bam and Bgcn, but not Nos, while Pum binding to *hb* mRNAs—the best-known targets of Pum and Nos—requires Nos. These results indicate that Pum uses distinct cofactors when binding different target mRNAs.

## Results

### Bam is required for repression through the *mad* 3′UTR

**Figure 1:**
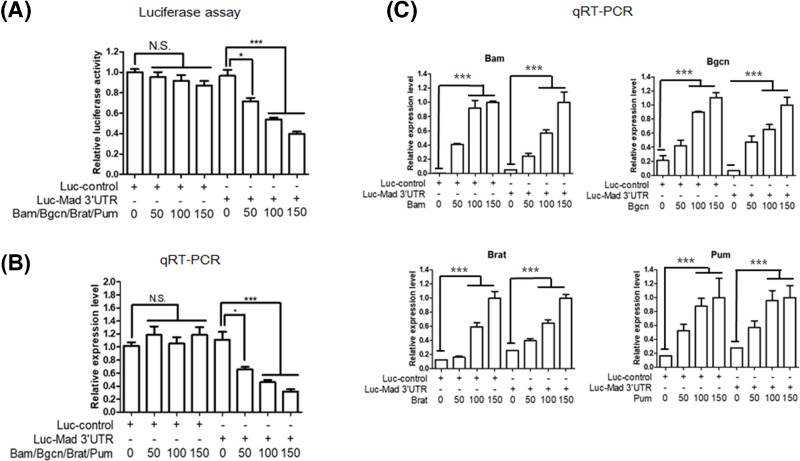
Dose dependency of repression (**A**–**C**) Control reporter *luc* and experimental reporter *luc-mad* 3′UTR FL (full-length) were co-transfected with varying quantities of vectors expressing Bam, Bgcn, Brat, and Pum (0, 50, 100, 150 ng). (A) Luciferase assays. The luciferase activity in the absence of expression vectors was set to 1.0. Data from three independent assays are represented as mean ± SEM. *P*-values ware determined by one-way ANOVA in GraphPad Prism 6.0, with *post-hoc* analysis using *Bonferroni’s* multiple comparison test. *P<0.05, ***P<0.001. (B) qRT-PCR assays. The mRNA levels of the *luc* control and *luc-mad* 3′UTR were measured by qRT-PCR. The value in the absence of expression vectors was set to 1.0. Data from three independent assays are represented as mean ± SEM. *P*-values ware determined by one-way ANOVA, with *post-hoc* analysis using *Bonferroni’s* multiple comparison test. *P<0.05, ***P<0.001. N.S. denotes non-specific. (C) qRT-PCR assays. The mRNA levels for *bam, bgcn, brat*, and *pum* were measured by qRT-PCR. The value in maximum expression vectors (150 ng/well) was set to 1.0. *P*-values ware determined by one-way ANOVA, with *post-hoc* analysis using *Bonferroni’s* multiple comparison test; ***P<0.001. Abbreviation: PCR, polymerase chain reaction.

Previously, it was shown that Bam and its obligate partner Bgcn physically interact with Pum and Brat [40,41]; this raises the possibility that Bam and Bgcn collaborate with Brat and Pum, which regulate *mad* at the post-transcriptional level through the *mad* 3′UTR in the absence of Nos. We examined this possibility using a reporter bearing the *mad* 3′UTR in Schneider’s 2 cells (S2 cells). Co-expression of all four factors (Bam, Bgcn, Brat, and Pum) did not affect expression of a control reporter lacking the *mad* 3′UTR, but greatly repressed *luciferase* (*luc*) expression in a dose-dependent manner from a *luc* reporter bearing the *mad* 3′UTR (Figure 1A). The level of *luc-mad* 3′UTR mRNAs decreased as expression of all four factors increased (Figure 1B). These results suggest that *luc* repression by the four factors is in part due to the destabilization of *luc-mad* 3′UTR mRNAs. Increasing the doses of expression vectors in the cells increased the expression of their corresponding genes (Figure 1C).

**Figure 2:**
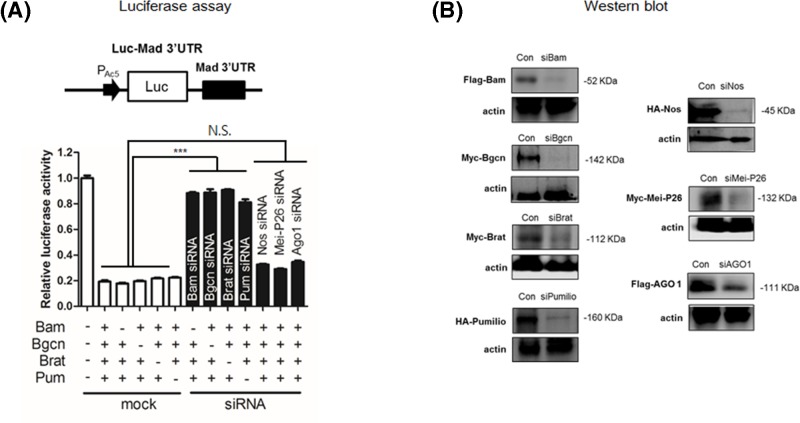
siRNA knockdown reporter assay (**A**) Luc reporter assays in cells transfected with *luc-mad 3*′*UTR* reporter, expression vectors, and siRNAs. The *luc* reporter was co-transfected with expression vectors and either scrambled siRNAs (denoted as mock) or gene-specific siRNAs (denoted as siRNA). + indicates presence of the expression vector while – indicates the absence of vectors. Luciferase activities of the *luc* reporter were normalized by the *β-galactosidase* activities of *pLacZ* as a transfection control, and in the absence of other expression vectors were set to 1.0. The mean ± SEM values were obtained from three independent experiments in triplicate. *P*-values ware determined by one-way ANOVA, with *post-hoc* analysis using *Bonferroni’s* multiple comparison test; ***P<0.001. N.S. denotes non-specific. (**B**) Knockdown of the target protein in cells treated with gene-specific siRNAs. S2 cells treated with either scrambled siRNA (con) or gene-specific siRNA and vectors expressing tagged proteins were subjected to Western blot analysis with anti-tag antibodies, showing knockdown of targeted proteins but not actin, by gene-specific siRNAs.

To determine whether all factors are required for repression, we omitted each expression vector individually. Interestingly, omitting any vector did not affect repression by the other three factors (Figure 2A). This could be ascribed to the presence of endogenous protein for repression. Each factor is indeed expressed in S2 cells (Supplementary Figure S1), making it necessary to remove the endogenous proteins. To identify siRNAs that can effectively knockdown target proteins, S2 cells were co-transfected with siRNAs and vectors expressing tagged proteins. For each gene, we tested two siRNAs and identified one that effectively reduced the target protein levels relative to control (scrambled) siRNAs (Figure 2B). We used these siRNAs to knockdown the corresponding endogenous protein in subsequent experiments.

Co-transfecting any three factors along with an siRNA for the fourth factor abolished repression (Figure 2A). These results indicate that each of Bam, Bgcn, Brat, and Pum is required for repression. In contrast, S2 cells treated with siRNAs specific for translational regulators Nos, Mei-P26, or Ago1 did not abrogate repression by four factors (Bam, Bgcn, Brat, and Pum) (Figure 2A). Taken together, these data show that Bam, Bgcn, Brat, and Pum, but not Nos, Mei-P26, or Ago1, are required for repression through the *mad* 3′UTR in S2 cells.

### Bam association with *mad* mRNA

**Figure 3:**
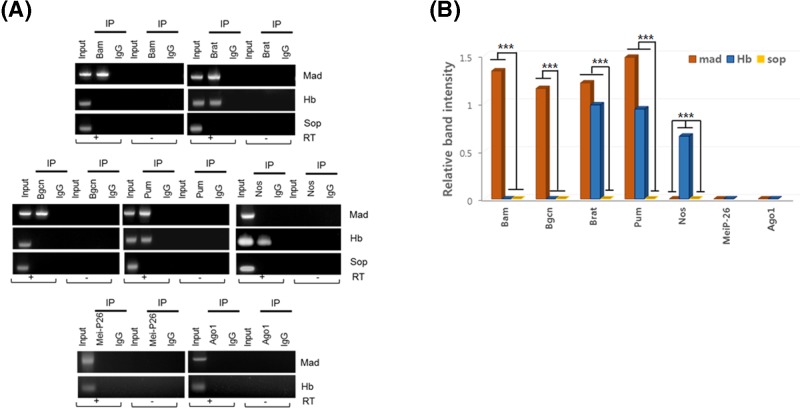
Protein–RNA association assay (**A**) Tagged proteins expressed in S2 cells were immunoprecipitated with either anti-tag antibodies or control IgG antibodies. The immunoprecipitates were subjected to RT in the presence (+) or absence (−) of reverse transcriptase, followed by PCR amplification with primers for *mad*, a positive control *hb*, and a negative control *sop*. Inputs were RNA extracts (1/10) prior to immunoprecipitation. Arepresentative agarose gel shows bands that indicate mRNAs associated with tagged Bam, Brat, Bgcn, Pum, Nos, Mei-P26, and Ago1 proteins in S2 cells. (**B**) Quantitation of RT-PCR band by ImageJ analysis (imagej.nih.gov/ij/). *P*-values from three independent data were determined by one-way ANOVA, with *post-hoc* analysis using *Bonferroni’s* multiple comparison test; ***P<0.001.

Next, we examined whether the four factors (Bam, Bgcn, Brat, and Pum) were associated with *mad* mRNA in S2 cells. Lysates from S2 cells individually expressing tagged-Bam, -Bgcn, -Brat, or -Pum were immunoprecipitated using tag antibodies. The immunoprecipitates were subjected to reverse transcription (RT) followed by polymerase chain reaction (PCR) amplification to determine association with *mad* mRNAs. *Hb* and *string-of-pearls* (*sop*) (also known as *ribosomal protein S2* (*rps2*)) mRNAs were used as a positive and a negative control, respectively. The results revealed that Bam, Bgcn, Brat, and Pum were all associated with *mad* mRNA (Figure 3), but not with the negative control *sop* mRNAs, which does not have PRE elements. Pum and Brat were associated with positive control *hb* mRNA, consistent with known interactions. In contrast, Bam and Bgcn were not associated with *hb* mRNA (Figure 3). The translational regulators Mei-P26 and Ago1 were not associated with either *mad* mRNA or *hb* mRNA (Figure 3). Nos was associated with *hb* mRNA as expected, but not with *mad* mRNA, which is consistent with Nos being required for *hb* repression but not *mad* repression [2,5,38] (Figure 3). Mock– immunoprecipitates with control antibodies (IgG) contained neither *mad* mRNA, *hb* mRNA, nor *sop* mRNA (Figure 3). Taken together, these results show that Bam and Bgcn are specifically associated with *mad* mRNA, but not *hb* mRNA, while Nos is specific to *hb* but not *mad* mRNA. Brat and Pum interact with both *mad* and *hb* mRNAs.

### Bam and Bgcn, but not Brat or Nos, are required for Pum binding to *mad* mRNA

**Figure 4:**
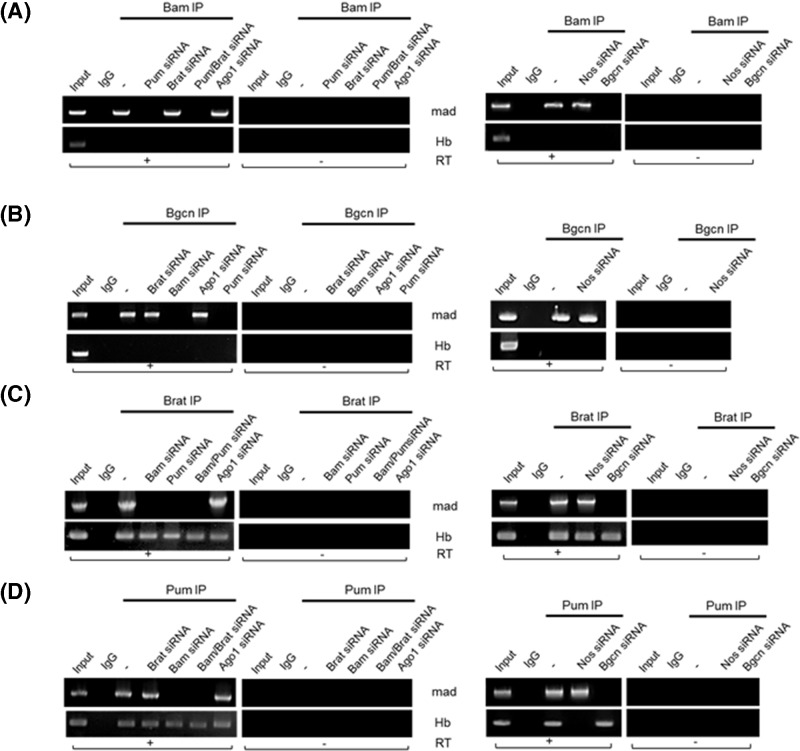
Protein–RNA association assay in cells treated with gene-specific siRNAs Tagged proteins were immunoprecipitated with tag antibodies from cells transfected with expression vectors and gene-specific siRNAs. – indicates control cells transfected with scrambled siRNAs. IgG indicates control experiments in which cell lysates were immunoprecipitated with control immunoglobulin. The immunoprecipitates were subjected to RT- PCR in the presence (+) or absence (−) of reverse transcriptase with primers specific to *mad* and *hb* mRNAs. Inputs were RNA extracts (1/10) prior to immunoprecipitation. A representative agarose gel shows bands that indicate mRNAs associated with tagged Bam (**A**), Bgcn (**B**), Brat (**C**), and Pum (**D**) proteins in S2 cells.

We were curious whether the association of Bam with *mad* mRNAs requires Pum. In lysates from control (scrambled) siRNA treated cells, Bam immunoprecipitates contained *mad* mRNA, but not *hb* mRNA (Figure 4A and Supplementary Figure S2). However, Bam immunoprecipitates of lysates from cells treated with Pum or Bgcn siRNA did not contain *mad* mRNA (Figure 4A and Supplementary Figure S2). In contrast, Bam immunoprecipitates of lysates from cells treated with Brat, Ago1, or Nos siRNA did contain *mad* mRNA (Figure 4A and Supplementary Figure S2). These demonstrate that the association of Bam with *mad* mRNA requires Pum and Bgcn, but not Brat, Ago1, or Nos. Similar experiments showed that Bgcn requires Bam and Pum, but not Brat, Ago, or Nos (Figure 4B and Supplementary Figure S2) in association with *mad* mRNAs. Meanwhile, Brat requires Bam, Pum, and Bgcn but not Ago1 or Nos when in association with *mad* mRNAs, and does not require other factors when binding *hb* mRNAs (Figure 4C and Supplementary Figure S2); the latter is consistent with a recent report that Brat can bind *hb* mRNAs by itself. Pum association with *hb* mRNAs does not require Bam, Bgcn, Brat, or Ago1 but does require Nos, while its association with *mad* mRNAs requires Bam and Bgcn, but not Brat or Nos (Figure 4D and Supplementary Figure S2). Taken together, the key conclusion is that Pum requires Bam and Nos for binding *mad* and *hb* mRNAs, respectively.

### Pum binding sites in the *mad* 3′UTR mediates repression and binding

**Figure 5:**
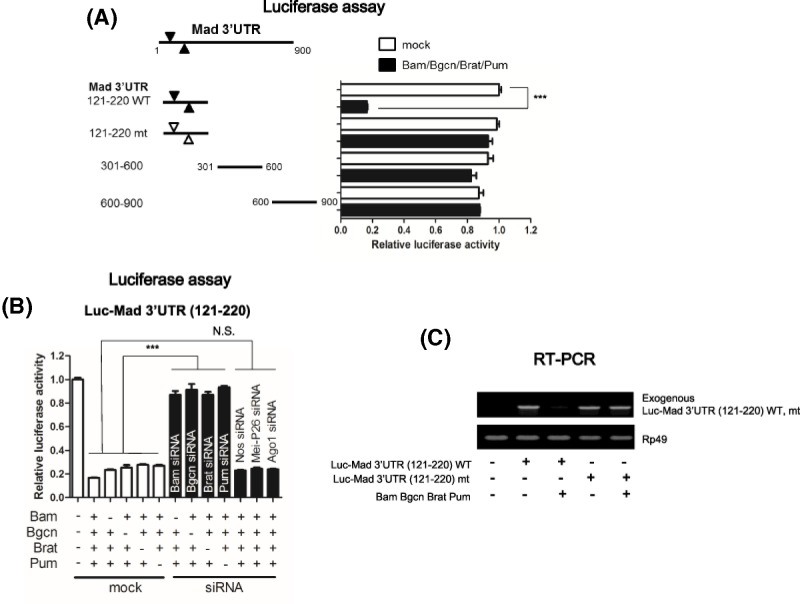
The UGUA sequence in the 121–220 region of *mad* 3′UTR mediates the repression of the *luc* reporter by Bam, Bgcn, Brat and Pum (**A**) Luciferase assays. The different regions of *mad* 3′UTR were fused to the *luciferase* coding sequence at the 3′UTR. UGUA (marked as filled triangle) present in the 121–220 region of the *mad* 3′UTR WT (wild-type) was mutated (mt) to ACUA (marked as empty triangle). The *luc* reporter vectors were co-transfected with expression vectors of Bam, Bgcn, Brat, and Pum. Luciferase activities of the *luc* reporters were normalized by the *β-galactosidase* activities of *pLacZ* as a transfection control. The *luciferase* activity of the *luc-mad* 3′UTR(121–220) in the absence of other expression vectors (denoted as mock) were set to 1.0. The mean ± SEM values were obtained from three independent experiments in triplicate. *P*-values ware determined by one-way ANOVA, with *post-hoc* analysis using *Bonferroni’s* multiple comparison test; ***P<0.001. (**B**) Luciferase assays. The *luc* reporter bearing 121–220 region of *mad* 3′UTR (Luc-Mad 3′UTR (121–220)) was transfected with expression vectors and either scrambled siRNAs (denoted as mock) or gene-specific siRNAs (denoted as siRNA). + indicates presence of the expression vector while – indicates absence of vectors. Luciferase activities of the *luc* reporter were normalized by the *β-galactosidase* activities of *pLacZ* as a transfection control, and in the absence of other expression vectors were set to 1.0. The mean ± SEM values were obtained from three independent experiments in triplicate. *P*-values ware determined by one-way ANOVA, with *post-hoc* analysis using *Bonferroni’s* multiple comparison test; ***P<0.001. N.S. denotes non-specific. (**C**) RT-PCR was performed to measure the levels of exogenous *luc-mad* 3′UTR [121-220] mRNAs and *luc-mad* 3′UTR [121–220]mt mRNAs in the absence or presence of Bam, Bgcn, Brat, and Pum. The PCR primers that detect exogenous *luc-mad* 3′UTR [121–220] and *luc-mad* 3′UTR [121–220] mt were used.

To identify the *cis*-element that mediates repression, *mad* 3′UTR was divided into three parts (1–300, 300–600, 600–900) and examined to see which region was responsible for the repression. The 1–300 region, but not the other regions, of *mad* 3′UTR-mediated repression of a reporter by Bam, Bgcn, Brat, and Pum (Figure 5A). We further narrowed the region to 121–220 which mediated the repression (Figure 5A). The 121–220 region contains two UGUA sequences, a core sequence found at many Pum-binding sites [26]. We mutated these two sites from **UG**UA to **AC**UA and examined whether the mutations affected the repression. Mutating both sequences abrogated the Bam, Bgcn, Brat, and Pum mediated repressions (Figure 5A), indicating that Bam, Bgcn, Pum, and Brat repressed translation through the UGUA sequence in the *mad* 3′UTR. Endogenous depletion of each of Bam, Bgcn, Brat, and Pum, but not of Nos, Meip-26, and Ago1 abrogated the repression of the reporter with a 121–220 region of the *mad* 3′UTR (Figure 5B). The RNA level of *luc-mad*, but not *luc-mad* mt (mutated), was reduced when Bam, Bgcn, Brat, and Pum were co-expressed (Figure 5C).

**Figure 6:**
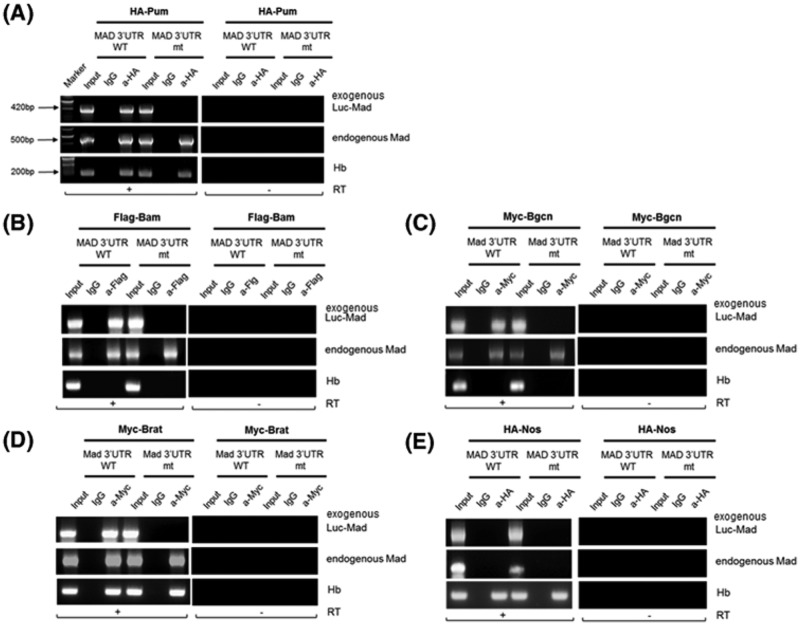
The UGUA sequence in the 121–220 region of the *mad* 3′UTR mediates the binding of Bam, Bgcn, Brat, and Pum to the exogenous *luc-mad* 3′UTR mRNAs RNA immunoprecipitation assays similar to Figure 4 were carried out to examine whether exogenous *luc-mad* 3′UTR [121–220] and *luc-mad* 3′UTR[121-220] mut mRNAs in which UGUA mutated to ACUA are associated with Bam, Bgcn, Brat, or Pum. PCR amplification primers detect exogenous *luc-mad* 3′UTR [121–220], endogenous *mad*, and, as a control, *hb*. Inputs were RNA extracts (1/10) prior to immunoprecipitation. A representative agarose gel shows bands that indicate mRNAs associated with tagged Pum (**A**), Bam (**B**), Bgcn (**C**), Brat (**D**) and Nos (**E**) proteins in S2 cells.

Next, we examined whether Bam, Bgcn, Brat, and Pum bound exogenous mRNAs of *luc* carrying 121–220 region of the *mad* 3′UTR (*luc-mad* 3′UTR[121-220]) but did not bind exogenous mRNAs of *luc-mad* 3′UTR [121–220] mt in which **UG**UA is mutated into **AC**UA. We carried out RNA immunoprecipitation assays in which we co-expressed either *luc-mad* 3′UTR [121–220] or *luc-mad* 3′UTR [121–220] mt and HA-tagged Pum in S2 cells. RT-PCR of Pum immunoprecipitates with HA antibodies revealed that Pum was associated with exogenous *luc-mad* 3′UTR [121–220] mRNAs, but not with *luc-mad* 3′UTR [121–220] mt mRNAs (Figure 6A). Pum-bound endogenous *mad* mRNAs and *hb* mRNAs. Mock-immunoprecipitates with nonspecific antibodies contained neither *luc-mad* 3′UTR [121–220] mRNAs nor *luc-mad* 3′UTR [121–220] mt mRNAs. Bam, Brat, and Bgcn bound *luc-mad* 3′UTR [121–220] mRNAs, but not *luc-mad* 3′UTR [121–220] mt mRNAs (Figure 6B–D). Nos protein bound neither *luc-mad* 3′UTR [121–220] mRNAs nor *luc-mad* 3′UTR [121–220] mt mRNAs (Figure 6E). Taken together, the UGUA sequence mediates binding of Bam, Bgcn, Brat, and Pum to *mad* 3′-UTR.

## Discussion

Recent evidence from *in vitro* assays indicates that the binding of Pum to target mRNAs requires Nos, suggesting that Nos plays an active role in Pum target binding [29,42]. However, other mRNAs associated with Pum were not dependent on Nos [29,42], suggesting that other factors might also play a role in the targeting of Pum to particular mRNAs. Here, we identify Bam and Bgcn as novel Pum cofactors in *mad* repression. Our data reveal that Bam, but not Nos, plays an active role in targeting Pum to *mad* mRNAs.

We found that nucleotides 121–220, but not other regions, of the *mad* 3′UTR are sufficient for repression. There are two putative Pum binding sites in this segment: 5′-**UGUA**CGGC-3′ and 5′-**UGUA**GGCC-3′ (hereafter, referred to as *mad*PREs). Except for four invariant nucleotides (i.e., **UGUA**) these imperfect PREs are distinct from the standard eight nucleotide Pum consensus binding sequence (i.e., 5′-**UGUAYAUA**-3′) [26]. The first two nucleotides (**UG**) are critical for Pum binding to *hb* mRNAs [23]. We show that mutating these nucleotides from **UG** to **AC** in *mad*PREs prevents repression and abolishes Bam, Bgcn, Pum, and Brat binding, demonstrating that these two *mad*PREs are essential *cis*-elements for *mad* repression and for the assembly of protein complexes on *mad* mRNAs.

Nos was recently shown to determine a subset of Pum targets, including *hb* mRNAs [41,42]. These Nos-Pum target mRNAs contain a Nos recognition sequence immediately upstream of a Pum binding sequence (5′-AAU **UGUANAUA**-3′ (AAU is the Nos binding sequence, the PRE is in bold)) [29]. Nos binds the three nucleotides to enhance Pum binding to *hb* mRNAs [29]. The sequence immediately upstream of the Pum binding sequence in *mad* mRNAs (*mad*PREs, UGA **UGUA**CGGC, GUC **UGUA**GGCC) does not contain the Nos recognition sequence, accounting for why Nos is not associated with *mad* mRNAs and why Nos is dispensable for Pum binding to and repression of *mad* mRNAs.

Brat is associated with both *hb* and *mad* mRNAs and is required for their repression [6,33,38]. Unlike Bam or Nos, Brat depletion does not abrogate Pum binding to *mad* and *hb* mRNA, suggesting Brat is not involved in the binding of Pum to its target mRNAs. In addition, we found that Brat has differential association with *mad* and *hb* mRNAs. Our *in vivo* binding assay showed that Brat associates with *hb* mRNA in the absence of other collaborators. This is consistent with recent data suggesting that Brat binds *hb* mRNA alone [33]. In contrast, Brat was not associated with *mad* mRNA in cells treated with siRNAs against Bam, Bgcn, or Pum. Given that Brat physically interacts with Bam and Pum, these results suggest that Brat is recruited to *mad* RNA indirectly via these interactions. The reason for this discrepancy between its binding to *hb* and *mad* mRNAs remains unclear. Although the mechanisms of Brat recruitment seem to differ, Brat binds to and is required for the repression of both *mad* and *hb* mRNAs. What could the role of Brat in repression be? Brat was recently shown to repress mRNAs independently of Pum and Nos [33]. Thus Brat is likely to increase the activity of repressive complexes formed on *cis*-elements, rather than being specifically involved in Pum targeting.

In conclusion, we propose that Pum targeting shifts depending on cofactors that are expressed differentially in cells. Given that Pum is associated with hundreds of mRNAs in *Drosophila* and thousands in *Caenorhabditis elegans* and mammals [25,26,43], while expression of Nos and Bam is more limited, we expect that there are as-yet-unidentified co-factors that specify Pum targets in different cells during embryonic development, during stem cell differentiation, and in the different subcellular regions of neurons.

## Materials and methods

### Chemicals and antibodies

All chemicals were obtained from Sigma–Aldrich. Primary antibodies were obtained as follows; anti-FLAG (M2; Sigma) (mouse), anti-Myc (rabbit) (Cell Signaling), and anti-HA (rat) (Roche Applied Science). All secondary antibodies were obtained from Roche Applied Science.

### S2 cells

The *Drosophila* S2 cells were maintained in a Shields and Sang M3 insect medium (Sigma–Aldrich) with 10% insect medium supplement (Sigma–Aldrich) and 1% antibiotics (HyClone) in a humidified atmosphere at 25°C.

### Vector constructions

The S2 cell expression vectors, pAc5.1A-FLAG-Bam, pAc5.1A-Myc-Bgcn, and pAc5.1A-HA-Pumilio were obtained from the previous studies [40]. pAc5.1A-Myc-Brat, pAc5.1A-HA-Nos, pAc5.1A-Myc-Mei-P26, and pAc5.1A-FLAG-Ago1 were from the previous studies [41].

### RNA immunoprecipitation assay

S2 cell extracts were generated from three six-well plates transiently transfected with the appropriate set of expression plasmids using the DDAB method [44]. The cleared extracts (6 mg) were mixed with 40 μl of anti-FLAG M2–conjugated agarose beads (Sigma) for precipitating Flag-tagged proteins incubated for overnight at 4°C. In case of HA or Myc tagged proteins, the extracts were mixed with 10 μl of anti-HA antibody (mouse) or 10 μl of anti-Myc antibody (mouse) for 16 h at 4°C followed by incubation with the 20 μl anti-IgA/G–conjugated agarose beads (Sigma) at 4°C overnight. For knockdown with siRNA, siRNAs were added in a concentration of 100 pmol siRNA/well and 1 μg expression plasmids/well of a six-well plate. Protocols after immunoprecipitation were described [45]. Different cycles (25–30) were then performed, and the resulting products were run on 1% agarose gels and visualized using EtBr. Primer sequence used for RT-PCR as follows: *mad* (forward, GACCTCGACGCCCTG; reverse, CACTTGAAATAGACTTGA), *hb* (forward, GTTCCCCATCACCATCAC; reverse, TGAAAGGTGGCTACAGTT), *sop* (forward, ACTCCTTACCAGGC;reverse, ACATTTAAAAGTTTATGAC).

### siRNA-induced knockdown

Synthetic siRNA was chemically manufactured (Bioneer). The synthetic duplexed oligonucleotides were diluted in DEPC water as working stock of 20 pmol/μl. All primary antibodies were used in a dilution of 1:3000 in 5% skimmed milk and all secondary antibodies were in a dilution of 1:5000 in 5% skimmed milk. To check whether proteins were knocked down by siRNA, S2 cells were transfected with expression vectors (1 μg/well) and siRNAs (100 pmol/well). S2 cells were transiently transfected with expression vectors, using the DDAB method [44]. Transfected cells harvested 72 h after transfection were washed in phosphate-buffered saline (HyClone Dulbecco’s Phosphate Buffer saline) and lysed with radioimmune precipitation assay buffer (50 mM Tris/HCl, pH 8.0, 150 mM NaCl, 1% Nonidet P-40, 5 mM EDTA, and 1 mM phenylmethylsulfonyl fluoride; ELPIS-Biotech.) containing protease inhibitors. The lysates were clarified by centrifugation at 13000 rpm (Eppendorf centrifuge) for 10 min at 4°C. The cleared extracts (3 mg) were mixed with 40 μl of anti-FLAG M2–conjugated agarose beads (Sigma–Aldrich) and rotated at 4°C overnight. The beads were precipitated by Eppendorf centrifugation and washed three times with washing buffer (20 mM HEPES (pH 7.7), 150 mM NaCl, 2.5 mM MgCl_2_, 0.05% Nonidet P-40, 10% glycerol, and 1 mM dithiothreitol) containing protease inhibitors. The bound proteins were eluted in 50 μl of 0.1 mM glycine acetate (pH 3.0) and precipitated by adding 10% (the final concentration) trichloroacetic acid and 1% (final) sodium deoxycholate. The elutes were incubated 30 min at −20°C and precipitated by centrifugation. The pellets were suspended in a 2× SDS loading buffer, and Western blot analysis was performed using anti-Flag, anti-HA and anti-Myc according to ECL protocol (Amersham Bioscience). The siRNA sequences were as follows: Pum sense siRNA- GGGAGAAAUCCGAUGGCAA; Nos sense siRNA-AAGAUUUUCCAAUUGCAGGAU; Brat sense siRNA-ACUGCUCAUGAGUUCAUGCAC; Bam sense siRNA-GCACGAUGCCUAAGAGCUUGA; Bgcn sense siRNA-GUUCCCGUGCACAUUCCGCAC; Ago1 sense siRNA-GACGGGCGGGCUACAAGCCCCA; Mei-P26 sense siRNA-UGAGACGGCCACGGUGGCGGA.

### Luciferase reporter assay and siRNA knockdown

The luciferase coding sequence was obtained from a pGL3-Basic vector (Promega), amplified by PCR, and cloned into the EcoRI/NotI sites pAc5.1/V5-His A (Invitrogen), generating pAc5.1A-LUC. The *Mad* 3′UTR were obtained from the RE72705 (DGRC, *Drosophila* Genomics Resource Center). Mad 3′UTR and its derivatives were cloned into XhoI/XbaI sites of pAC5.1A-LUC to generate pAC5.1A-LUC-*Mad* 3′UTR, pAC5.1A-LUC- *Mad* 3′UTR (1–300) bp, pAC5.1A-LUC-*Mad* 3′UTR (121–220) bp pAC5.1A-LUC-*Mad* 3′UTR (301–600), and pAC5.1A-LUC- *Mad* 3′UTR (601–900). For the generation of LUC-*Mad* 3′UTR (121–220) mutant construct, both the UGUA sequences, in the region of (140–143 and 194–197 nucleotides) were mutated to ACUA sequences., using the primers which were designed from region of 121–220 nucleotides of *Mad* 3′UTR. The LUC-*Mad* 3′UTR mutant was synthesized from RE72705, with forward primer containing first UGUA site mutated to ACUA sequence and reverse primer containing another UGUA site mutated to ACUA sequence which was further cloned into XhoI/XbaI sites of pAC5.1A-LUC vector. The primer sequences used for PCR (*mad* 3′UTRmutant) are as follows: forward, AATGGAGAGAGCATAGGTCACTAGGCC; reverse, GGCGGGTATTGGAGTAGCTGCCGT ACATCATCTAT. All the constructs were verified by DNA sequencing. The siRNAs were used for Luciferase reporter assay in a concentration of 50 pmol siRNAs/well of a 24-well plate. pAc5.1A-LUC/ *Mad* 3′UTR reporter (100 ng/well), pAc5.1A-LacZ plasmid (400 ng/well), and the expression plasmids (200 ng/well) were co- transfected using the DDAB method [44]. The transfected cells were plated into a 24-well plate and harvested and assayed 72 h post-transfection for luciferase activity. The luciferase activities were measured and normalized to *β-galactosidase* activity. These results were obtained from triplicate samples, and the data are representative of a minimum three- to- five independent experiments.

### RT-PCR and qRT-PCR

Total RNA was isolated using TRI Reagent (Molecular Research Center) in transfected S2 cells according to the manufacturer’s instruction. RNA was reverse-transcribed into cDNA using TOP Script™ RT DryMIX (Enzynomics) and Oligo dT. Briefly, the reaction mixture contained 1 μg of sample RNA, 100 pmoles of Oligo dT (18-mer), and the TOP Script RT premix. The reaction consisted of incubation at 42°C for 5 min, 50°C for 60 min and 94°C for 5 min for RT. Then 1 µl of cDNA used for RT-PCR using PCR premix-nTaq (Enzynomics) with 10 pmoles of each forward and reverse PCR primer. The reaction consisted of incubation at 94°C for 5 min followed by 22 cycles of 94°C for 10 s, 57°C for 10 s and 72°C for 10 s. Ten microliters of the amplified reaction was electrophoresed on a 2% agarose gel followed by staining with Ethidium Bromide and imaging. Real-Time PCR was performed on a StepOnePlus Real-Time PCR System (Thermo Fisher Scientific) using TOPreal™ qPCR 2X PreMIX (SYBR Green with high ROX) kit (Enzynomics). RP49 was used as a reference gene to normalize each gene. *bam* (forward, GATTTCGAGAAATACGACGAGTG; reverse, CGCAGACCAATTAGCAATCT), *bgcn* (forward, GCACAGAGCTTCCGCACA; reverse, AGATCCATTCGTCTATAAAGACGT), *brat* (forward, GCCCACTACAATCCCTACGA; reverse, CGGATAGATAGTGGCCGAAA), *pum* (forward, TACTCGCTAAGCACCCATCC; reverse, CCTTCATCATCACGTGCAAC), Luc (forward, CTCATAAAGGCCAAGAAGGG; reverse, TAGAAGGCACAGTCGAGG), Luc-*Mad* 3′UTR (forward, ATGACGCCGGTGAAC; reverse, TCACTCACACGCACTCATCA).

### Statistical analysis

Luciferase assays and qRT-PCR were performed in triplicate at least three times. Data calculation and statistical analysis were performed using GraphPad Prism 6.0 software. *P*-values ware determined by one-way ANOVA in GraphPad Prism 6.0, with post-hoc analysis using Bonferroni’s multiple comparison test.

## Supporting information

**Supplementary Figure S1:** (A) Agarose gel image of RT-PCR of S2 cells. Endogenous mRNA levels for Bam, Bgcn, Brat, Pum and Nos in S2 cells were measured by RT-PCR. PCR performed at 57 °C with 35 cycles produced DNA bands of the expected sizes, showing that S2 cells express endogenous Bam, Bgcn, Brat, Pum, and Nos proteins. C denotes cDNA; g denotes genomic DNA. Cell confluency: (1) 70%, (2) 100%, (3) >100%.

**Supplementary Figure S2:** Quantification of RT-PCR band intensities by image J analysis (imagej.nih.gov/ij/). P-values from three independent data were determined by one-way ANOVA, with *post-hoc* analysis using *Bonferroni’s* multiple comparison test. ***p < 0.001.

## Abbreviations

Bam: bag-of-marbles
Brat: brain tumor
hb: *hunchback*
mad: *mothers against dpp*
NHL: NCL-1, HT2A, and LIN-41
NRE: Nanos response element
PCR: polymerase chain reaction
PRE: Pumilio response element
RT: reverse transcription
S2 cell: Schneider’s 2 cell
