# Supplementary material for: The target specificity of the RNA binding protein Pumilio is determined by distinct co-factors
Source: Biosci Rep. 2019 Jun 4;39(6):BSR20190099. doi: 10.1042/BSR20190099 (PMC6549094; doi:10.1042/BSR20190099)
Supplement: Supplementary file 1 [file bsr20190099_Supp1.pdf]

Fig S1

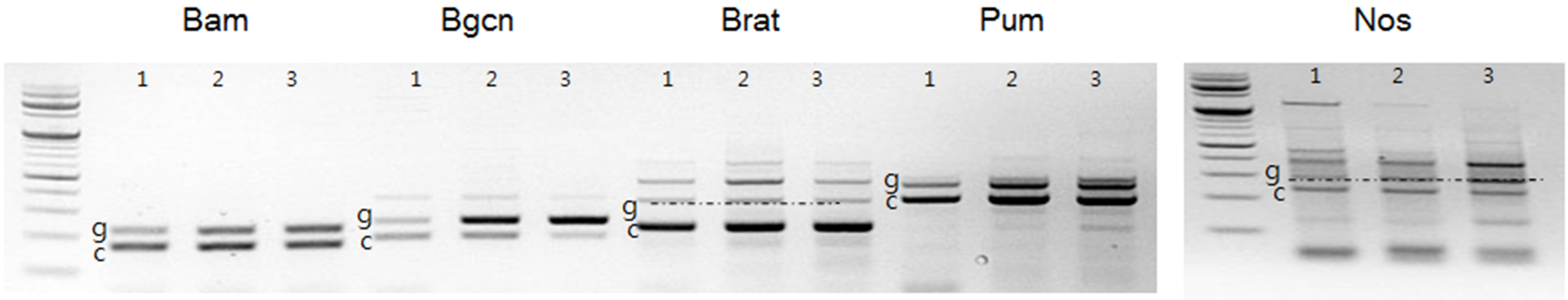

Fig S2

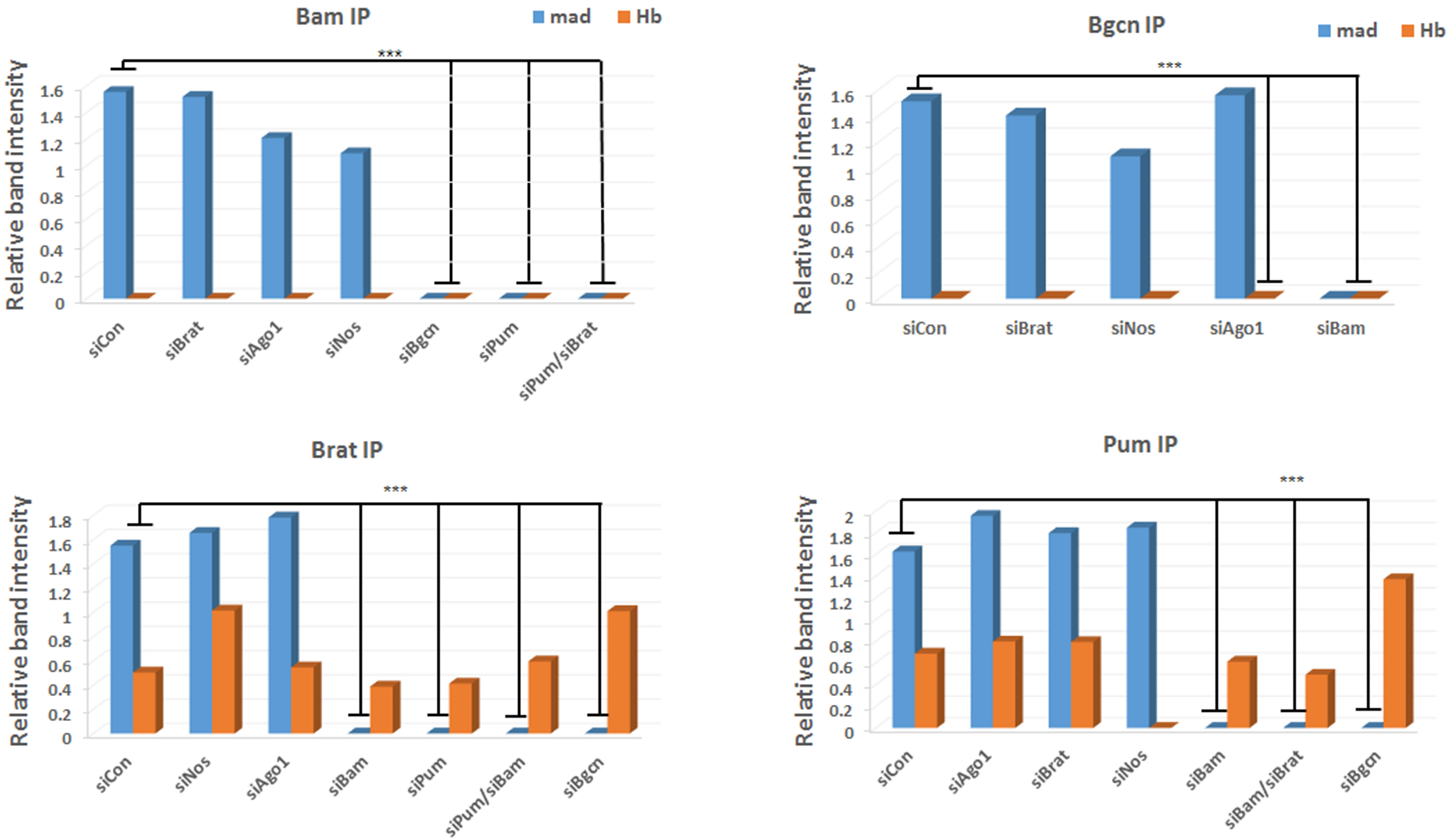
Bam IP
mad
Hb
***
Relative band intensity
0
0.2
0.4
0.6
0.8
1
1.2
1.4
1.6
siCon
siBrat
siAgo1
siNos
siBgcn
siPum
siPum/siBrat
Bgcn IP
mad
Hb
***
Relative band intensity
0
0.2
0.4
0.6
0.8
1
1.2
1.4
1.6
siCon
siBrat
siNos
siAgo1
siBam
Brat IP
***
Relative band intensity
0
0.2
0.4
0.6
0.8
1
1.2
1.4
1.6
1.8
siCon
siNos
siAgo1
siBam
siPum
siPum/siBam
siBgcn
Pum IP
***
Relative band intensity
0
0.2
0.4
0.6
0.8
1
1.2
1.4
1.6
1.8
2
siCon
siAgo1
siBrat
siNos
siBam
siBam/siBrat
siBgcn

Supporting Information

Figure S1

(A) Agarose gel image of RT-PCR of S2 cells. Endogenous mRNA levels for Bam, Bgcn, Brat, Pum and Nos in S2 cells were measured by RT-PCR. PCR performed at 57 °C with 35 cycles produced DNA bands of the expected sizes, showing that S2 cells express endogenous Bam, Bgcn, Brat, Pum, and Nos proteins. C denotes cDNA; g denotes genomic DNA. Cell confluency: (1) 70%, (2) 100%, (3) >100%.

Figure S2

Quantification of RT-PCR band intensities by image J analysis (imagej.nih.gov/ij/). P-values from three independent data were determined by one-way ANOVA, with *post-hoc* analysis using *Bonferroni's* multiple comparison test. *** $p < 0.001$.
